# Outcomes of 6‐mm diameter fully covered self‐expandable metal stents for preoperative biliary drainage in pancreatic cancer

**DOI:** 10.1002/deo2.360

**Published:** 2024-04-09

**Authors:** Hiroki Nakagawa, Tsuyoshi Takeda, Takeshi Okamoto, Takafumi Mie, Akiyoshi Kasuga, Takashi Sasaki, Masato Ozaka, Takahisa Matsuda, Yoshinori Igarashi, Naoki Sasahira

**Affiliations:** ^1^ Department of Hepato‐Biliary‐Pancreatic Medicine Cancer Institute Hospital of Japanese Foundation for Cancer Research Tokyo Japan; ^2^ Department of Internal Medicine Division of Gastroenterology and Hepatology, Omori Medical Center Toho University Tokyo Japan

**Keywords:** distal malignant biliary obstruction, neoadjuvant therapy, pancreatic cancer, pancreatitis, preoperative biliary drainage

## Abstract

**Background:**

10‐mm self‐expandable metal stents (SEMSs) are commonly used for preoperative biliary drainage in pancreatic cancer. However, smaller diameter SEMSs have attracted attention with the attempt to reduce stent‐related adverse events (AEs).

**Methods:**

We retrospectively analyzed consecutive borderline resectable pancreatic cancer patients who underwent neoadjuvant therapy and fully covered SEMS (FCSEMS) placement from April 2015 to May 2023. The primary outcome was stent‐related non‐event rate (NER), which was defined as the rate of completion of surgery without developing any preoperative events (recurrent biliary obstruction [RBO] or stent‐related AEs). Secondary outcomes included stent‐related AEs, causes of RBO, and cumulative incidence of RBO. Risk factors for pancreatitis, RBO, and stent migration were also examined.

**Results:**

A total of 76 patients were included (6‐mm group: 23; 10‐mm group: 53). Stent‐related NER (57% vs. 64%, *p* = 0.610), stent‐related AEs (4% vs. 15%, *p* = 0.263), overall RBO rates (39% vs. 23%, *p* = 0.168), cumulative incidence of RBO (hazard ratio, 2.24; 95% confidence interval, 0.95–5.25; *p* = 0.065) were not significantly different between the two groups. Tumor involvement of the pancreatic duct was identified as a risk‐reducing factor for pancreatitis, while an FCSEMS diameter of 6 mm was not identified as a risk factor for RBO and stent migration.

**Conclusions:**

Stent‐related NER was not significantly affected by FCSEMS diameter. Further studies are needed to confirm the usefulness of 6‐mm diameter FCSEMS for preoperative biliary drainage in patients with borderline resectable pancreatic cancer.

## INTRODUCTION

Neoadjuvant therapy (NAT) has been accepted as the standard treatment for resectable and borderline resectable (BR) pancreatic cancer (PC) due to higher margin‐negative resection rates and overall survival compared to upfront surgery.^1^, ^2^, ^3^, ^4^, ^5^ Preoperative biliary drainage (PBD) for such patients is becoming increasingly important due to the resulting prolongation of the preoperative waiting period. Several studies have reported superior stent patency of self‐expandable metal stents (SEMS) compared to plastic stents for PBD,^6^, ^7^, ^8^ with the European Society of Gastrointestinal Endoscopy Clinical Guideline recommending SEMSs with 10‐mm diameters.^9^

A recent meta‐analysis of patients with PC who underwent NAT found that SEMSs are associated with a reduced risk of recurrent biliary obstruction (RBO), need for reintervention, and delay in NAT when compared to plastic stents, despite a higher risk of stent‐related complications including cholecystitis and pancreatitis.^10^ As cholecystitis and pancreatitis may lead to delays in NAT and surgery, it is important to avoid not only RBO but also stent‐related adverse events (AEs) before surgery. We previously reported that pancreatitis may be more common in BR PC compared to unresectable PC when a 10‐mm diameter fully covered SEMS (FCSEMS) was used for distal malignant biliary obstruction (MBO).^11^ Recently, smaller diameter SEMSs have attracted attention, due to the perceived possibility of reducing stent‐related AEs. To date, only two studies have compared the efficacy and safety of SEMSs with 6‐mm diameters to plastic stents or SEMSs with 10‐mm diameters.^12^, ^13^

Therefore, we conducted this retrospective study to evaluate the efficacy and safety of 6‐mm FCSEMSs compared to 10‐mm FCSEMSs for PBD in patients with BR PC.

## METHODS

### Patients

Consecutive patients with BR pancreatic head cancer who underwent NAT and FCSEMS placement at our institution between April 2015 and May 2023 were identified from our prospectively maintained database. Excluded patients were as follows: (1) patients who received more than one biliary SEMS, (2) patients who received a SEMS above the papilla, (3) patients with a concomitant hilar biliary obstruction, (4) patients with surgically altered anatomy, (5) patients who had a history of pancreatic resection, (6) patients who refused or were unfit for surgery at the time of diagnosis, and (7) patients who were transferred to another hospital during NAT. The type of SEMS used was mainly determined by the time period during which the patient underwent SEMS placement. In general, FCSEMS with 10 mm diameters (10‐mm FCSEMS) were used between April 2015 and December 2020, while FCSEMS with 6 mm diameters (6‐mm FCSEMS) was used between January 2021 and May 2023. This study was conducted in accordance with the Declaration of Helsinki and was approved by our Institutional Review Board (approval number: 2022‐GB‐080). Study participants were offered an opportunity to opt out of this study for any reason.

### Endoscopic procedures

Endoscopic retrograde cholangiopancreatography (ERCP) was performed using a therapeutic duodenoscope (JF‐260V, TJF‐260V, or TJF‐Q290V; Olympus Medical Systems) under conscious sedation with pethidine and midazolam. Endoscopic sphincterotomy (EST) with a small to moderate‐sized incision was performed before SEMS placement in most patients. A SEMS was generally placed at least 2 cm below the bifurcation of the hepatic duct in order to achieve a successful biliary anastomosis during surgery. The length of SEMSs was determined based on cholangiographic findings. Prophylactic rectal nonsteroidal anti‐inflammatory drugs (NSAIDs) use and/or pancreatic duct stent placement were left to the endoscopist's discretion. Diclofenac suppository was administered after the conclusion of ERCP. The dose of diclofenac was generally determined based on the patient's weight. Dose reduction was considered in some patients including those with older age and renal dysfunction at the endoscopist's discretion. All procedures were performed by experienced pancreaticobiliary endoscopists or by trainees under their direct supervision.

The 6‐mm FCSEMSs used in this study were HANAROSTENT Biliary Full Cover Benefit and HANAROSTENT Biliary Full Cover (M.I. Tech). The 10‐mm FCSEMSs used in this study were HANAROSTENT Biliary Full Cover (M.I. Tech), HANAROSTENT Biliary Full Cover NEO (M.I. Tech), Evolution Biliary Controlled‐Release Stent Fully Covered (Cook Medical), and Niti‐S SUPREMO‐10 stent (TaeWoong Medical). The pancreatic duct stent used in this study was Geenen Pancreatic Stent Sets (Cook Medical).

### Neoadjuvant chemotherapy

Patients generally received four cycles (16 weeks) of gemcitabine plus nab‐paclitaxel (GnP) combination therapy or eight cycles (16 weeks) of modified FOLFIRINOX (mFFX). The majority of patients received GnP, while a minority received mFFX due to a temporary shortage of nab‐paclitaxel in Japan beginning in August 2021.

Computed tomography and magnetic resonance imaging were generally performed for restaging after completion of NAT. The final decision to perform surgical exploration was determined by an expert panel consisting of medical oncologists, surgeons, and radiologists.

### Outcome measurements and definitions

The primary outcome was stent‐related non‐event rate (NER), which was defined as the rate of completion of surgery without preoperative events, which were in turn defined as RBO or stent‐related AEs before surgery. For patients who could not receive surgery due to unresectability following NAT, stent‐related NER was defined as the rate of developing none of the above‐mentioned events until the diagnosis of unresectability. Secondary outcomes were technical success, functional success, stent‐related AEs, causes of RBO, cumulative incidence of RBO, and postoperative complications. Relevant outcomes were generally evaluated according to Tokyo Criteria 2014.^14^ Technical success was defined as the successful deployment of a SEMS at the intended location, while functional success was defined as a 50% decrease in, or normalization of, serum bilirubin level within 14 days after SEMS placement. Asymptomatic stent migration, which did not require reintervention, was not considered to be stent‐related AEs or preoperative events. Stent‐related AEs were defined according to Tokyo Criteria 2014^14^ and the severity of AEs was graded according to the American Society of Gastrointestinal Endoscopy lexicon guidelines.^15^ RBO was defined as stent occlusion, stent migration, and non‐occlusion cholangitis requiring endoscopic reintervention in this study. Postoperative complications were graded according to the Clavien‐Dindo classification system.^16^ Pancreatic volume index was calculated as the sum of lengths of the normal pancreatic parenchyma measured at three sections (the level of aorta, left adrenal gland, and left kidney), as reported in our previous study.^11^ The Preoperative waiting period was defined as the period from SEMS placement until surgery or the diagnosis of unresectability after commencing NAT. Tumor involvement of the pancreatic duct was defined as main pancreatic duct obstruction with upstream dilation. Tumor involvement of the orifice of the cystic duct was defined as tumor extension around the orifice of the cystic duct evaluated by either or both computed tomography and magnetic resonance cholangiopancreatography. Follow‐up data were confirmed until September 30, 2023.

### Statistical analysis

Continuous variables are expressed as median (interquartile range) and compared using the Mann‐Whitney U test. Categorical variables are expressed as absolute numbers (proportions) and analyzed using either the *χ* ^2^ test or Fisher's exact test as appropriate. The cumulative incidence of RBO was estimated using competing risk analysis, displayed graphically as cumulative incidence curves, and compared using Gray's test. Asymptomatic complete outward migration, stent removal due to AEs or surgery, and death without RBO were considered competing events. Logistic regression analysis was performed to evaluate risk factors for pancreatitis, RBO, and stent migration. Factors with *p* < 0.20 were included in the multivariate analysis. *p*‐Values < 0.05 were considered statistically significant. Statistical analyses were performed using EZR software version 1.54.^17^

## RESULTS

### Patient characteristics

**FIGURE 1:**
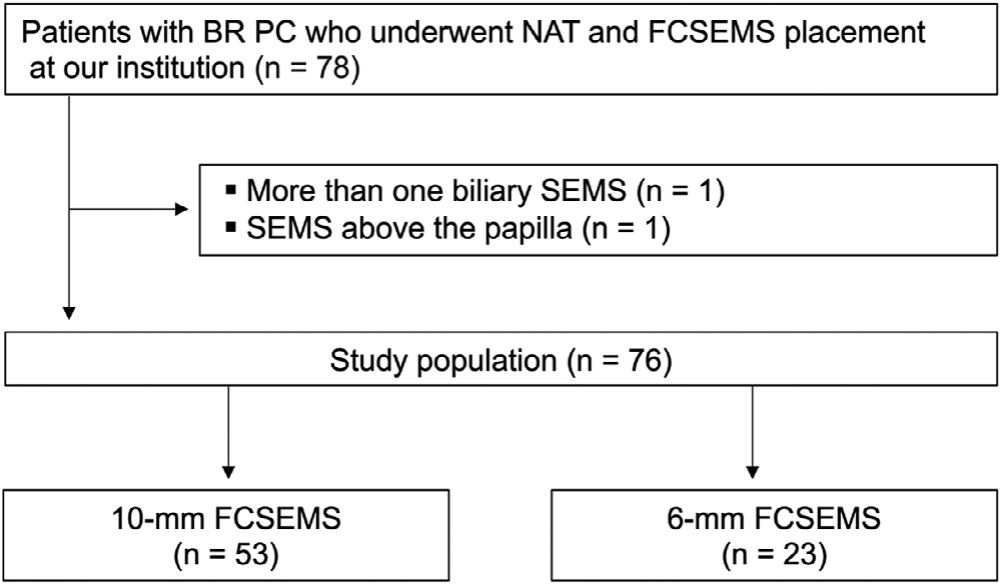
Patient flowchart. BR, borderline resectable; PC, pancreatic cancer; NAT, neoadjuvant therapy; FCSEMS, fully covered self‐expandable metal stent; SEMS, self‐expandable metal stent.

A total of 78 patients underwent NAT for BR PC and FCSEMS placement for distal MBO at our institution between April 2015 and May 2023. Two patients were excluded and the remaining 76 patients were analyzed (Figure 1). Twenty‐three patients were treated with 6‐mm FCSEMS and 53 were treated with 10‐mm FCSEMS. Patient characteristics are shown in Table 1. Modified FOLFIRINOX was more commonly used in the 6‐mm group (35% vs. 2%, *p* < 0.001), while GnP was more commonly used in the 10‐mm group (65% vs. 98%, *p* < 0.001). A higher proportion of patients in the 6‐mm group received prior biliary drainage with a plastic stent (43% vs. 17%, *p* = 0.021); 16 patients had previously undergone plastic stent placement at the referring hospital, and three patients at our hospital. The median preoperative waiting period was significantly shorter in the 6‐mm group (111 vs. 133 days, *p* = 0.028). Other parameters were not significantly different between the two groups.

**TABLE 1:** Baseline characteristics.

	6‐mm FCSEMS *n* = 23	10‐mm FCSEMS *n* = 53	*p‐*value
Age, years	65.0 (59.5–73.0)	68.0 (61.0–74.0)	0.606
Sex, male	10 (44%)	27 (51%)	0.622
ECOG PS, 0/1	21 (91%) /2 (9%)	49 (92%) /4 (8%)	>0.999
Primary tumor size, mm	27.0 (24.0–32.0)	29.0 (26.0–34.0)	0.248
Neoadjuvant therapy			<0.001
GnP	15 (65%)	52 (98%)	
mFFX	8 (35%)	1 (2%)	
Post‐cholecystectomy	1 (4%)	6 (11%)	0.668
Tumor involvement of the pancreatic duct	19 (83%)	48 (91%)	0.441
Tumor involvement of the orifice of the cystic duct	0 (0%)	0 (0%)	N/A
Pancreatic volume index, mm	32.3 (25.1–46.2)	30.6 (22.5–36.5)	0.091
Prior biliary drainage with plastic stent	10 (43%)	9 (19%)	0.021
Preoperative waiting period, days	111 (80–133)	133 (114–151)	0.028

Continuous variables are expressed as median (interquartile range) and categorical variables are expressed as absolute numbers (proportions).

Abbreviations: ECOG, Eastern Cooperative Oncology Group; FCSEMS, fully covered self‐expandable metal stent; GnP, gemcitabine plus nab‐paclitaxel; mFFX, modified FOLFIRINOX; N/A, not available; PS, performance status.

Details of ERCP procedures are shown in Table 2. The technical success rate was 100% in both groups. EST was performed in all but two patients in the 6‐mm group (for technical reasons: 1, due to the endoscopist's discretion: 1). All patients in the 10‐mm group underwent EST. Two patients in the 6‐mm group received pancreatic stents due to concerns for post‐ERCP pancreatitis. One patient received a 5 Fr pancreatic stent, while the other patient received a 7 Fr pancreatic stent. The use of prophylactic rectal NSAIDs was significantly higher in the 6‐mm group (87% vs. 49%, *p* = 0.002).

**TABLE 2:** Details of endoscopic retrograde cholangiopancreatography procedures.

	6‐mm FCSEMS *n* = 23	10‐mm FCSEMS *n* = 53	*p‐*value
Technical success	23 (100%)	53 (100%)	N/A
FCSEMS placement before NAT	12 (52%)	41 (77%)	0.055
EST	21 (91%)	53 (100%)	0.089
FCSEMS length			0.435
4–6 cm	13 (57%)	36 (68%)	
7–8 cm	10 (44%)	17 (32%)	
Pancreatic stent placement	2 (9%)	0 (0%)	0.089
Prophylactic rectal NSAIDs use	20 (87%)	26 (49%)	0.002
25 mg	4 (17%)	16 (30%)	
50 mg	16 (70%)	10 (19%)	

Categorical variables are expressed as absolute numbers (proportions).

Abbreviations: EST, endoscopic sphincterotomy; FCSEMS, fully covered self‐expandable metal stent; N/A, not available; NAT, neoadjuvant therapy; NSAIDs, nonsteroidal anti‐inflammatory drugs.

### Outcome measures

The outcomes of PBD with FCSEMS are shown in Table 3. The functional success rate was 96% in the 6‐mm group and 94% in the 10‐mm group (*p* > 0.999). Overall stent‐related AEs were not significantly different between the two groups (4% vs. 15%, *p* = 0.263). Pancreatitis occurred in 4% of patients in the 6‐mm group and in 13% of patients in the 10‐mm group (*p* = 0.422). Two of the seven patients who developed pancreatitis in the 10‐mm group experienced treatment delays. One patient required long‐term hospitalization due to severe pancreatitis, which led to a delay in NAT initiation. The other patient required an extended duration of NAT (delay in surgery) because it was difficult to differentiate between inflammation caused by pancreatitis and tumor invasion. Overall RBO rates were 39% in the 6‐mm group and 23% in the 10‐mm group (*p* = 0.168). Stent‐related NER was not significantly different between the two groups (57% vs. 64%, *p* = 0.610).

**TABLE 3:** Outcomes of preoperative biliary drainage with fully covered self‐expandable metal stent.

	6‐mm FCSEMS *n* = 23	10‐mm FCSEMS *n* = 53	*p‐*value
Functional success	22 (96%)	50 (94%)	>0.999
Stent‐related AEs^a^	1 (4%)	8 (15%)	0.263
Pancreatitis	1 (4%)	7 (13%)	0.422
Mild/moderate/severe	0/1/0	1/4/2	
Cholecystitis	0 (0%)	1 (2%)	>0.999
Mild/moderate/severe	0/0/0	0/1/0	
Abscess around the bile duct	0 (0%)	1 (2%)	>0.999
RBO	9 (39%)	12 (23%)	0.168
Occlusion	4 (17%)	4 (8%)	0.234
Biliary sludge/food impaction	3	4	
Kinking	1	0	
Migration	4 (17%)	4 (8%)	0.234
Inward	2	1	
Outward	2	3	
Non‐occlusion cholangitis	1 (4%)	4 (8%)	>0.999
Stent‐related NER^b^	13 (57%)	34 (64%)	0.610

Categorical variables are expressed as absolute numbers (proportions).

Abbreviations: AEs, adverse events; FCSEMS, fully covered self‐expandable metal stent; NER, non‐event rate; RBO, recurrent biliary obstruction.

^a^ One patient in the 10‐mm group developed both pancreatitis and cholecystitis at different times.

^b^ Stent‐related NER was defined as the rate of completion of surgery without developing any preoperative events including RBO and stent‐related AEs for patients who underwent surgery, while it was defined as the rate of developing none of the above‐mentioned events until the diagnosis of unresectability for patients who did not undergo surgery.

**FIGURE 2:**
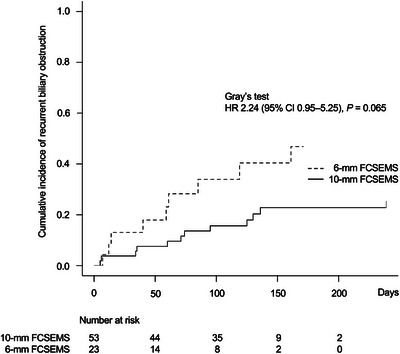
Cumulative incidence of recurrent biliary obstruction, stratified by stent group. HR, hazard ratio; CI, confidence interval; FCSEMS, fully covered self‐expandable metal stent.

The cumulative incidence of RBO was not significantly different between the two groups (hazard ratio [HR], 2.24; 95% confidence interval [CI], 0.95–5.25; *p* = 0.065; Figure 2).

The outcomes of neoadjuvant therapy and surgery are shown in Table 4. The percentage of patients who showed a reduction in primary tumor size was 57% in the 6‐mm group and 79% in the 10‐mm group (*p* = 0.054). The resection rate was not significantly different between the two groups (52% vs. 74%, *p* = 0.101). Causes of unresectability were disease progression in 23 patients (distant metastasis: 12, local progression: 11) and death in one patient due to NAT‐related AE. Postoperative complications (≥ Clavien‐Dindo grade 3) were not significantly different between the two groups (9% vs. 10%, *p* > 0.999).

**TABLE 4:** Outcomes of neoadjuvant therapy and surgery.

	6‐mm FCSEMS *n* = 23	10‐mm FCSEMS *n* = 53	*p‐*value
The reduction rate of primary tumor			
≥ 30%	4 (17%)	15 (28%)	0.395
0%–30%	9 (39%)	27 (51%)	0.454
˗20%–0%	7 (30%)	7 (13%)	0.107
< ˗20%	3 (13%)	4 (8%)	0.426
Reduction of the primary tumor	13 (57%)	42 (79%)	0.054
Resection rate^a^	11 (52%)	39 (74%)	0.101
Causes of unresectability^a^			
Disease progression	10 (48%)	13 (25%)	0.093
Distant metastasis	6 (29%)	6 (11%)	0.087
Local progression	4 (19%)	7 (13%)	0.495
Death	0 (0%)	1 (2%)	>0.999
Postoperative complication^b^			
≥ Clavien‐Dindo grade 3^c^	1 (9%)	4 (10%)	>0.999
Pancreatic fistula	0	1	
Surgical site infection	1	1	
Liver failure	0	1	
Chylous ascites	0	1	
Postoperative hemorrhage	0	1	
Pleural effusion	0	1	
Bile leakage	0	0	

Categorical variables are expressed as absolute numbers (proportions).

Abbreviation: FCSEMS, fully covered self‐expandable metal stent.

^a^ Denominators adjusted to exclude two patients who refused surgery in the 6‐mm group.

^b^ Denominators adjusted to exclude 26 patients who did not undergo surgery (12 patients and 14 patients in the 6‐ and 10‐mm groups, respectively).

^c^ Two patients in the 10‐mm group developed multiple complications (pancreatic fistula and surgical site infection: 1; liver failure and chylous ascites: 1).

### Risk factors for pancreatitis, RBO, and stent migration

Univariate and multivariate logistic regression analyses of risk factors for pancreatitis, RBO, and stent migration are shown in Table 5. Tumor involvement of the pancreatic duct (odds ratio, 0.13; 95 % CI, 0.02–0.78; *p* = 0.025) was identified as a risk‐reducing factor for pancreatitis, while none of the parameters including FCSEMS diameter were identified as risk factors for RBO and stent migration.

**TABLE 5:** Univariate and multivariate logistic regression analyses of risk factors for pancreatitis, recurrent biliary obstruction, and stent migration.

		Univariate			Multivariate		
		Odds ratio	95 % CI	*p‐*Value	Odds ratio	95 % CI	*p‐*value
Pancreatitis							
Age	<50 years	1.80	0.18–17.7	0.614			
Sex	Female	0.94	0.22–4.08	0.937			
Tumor involvement of the pancreatic duct	Yes	0.16	0.03–0.85	0.031	0.13	0.02–0.78	0.025
Pancreatic volume index	≥31	1.00	0.23–4.33	> 0.999			
FCSEMS diameter	6 mm	0.30	0.03–2.58	0.272			
FCSEMS length	≥7 cm	1.96	0.45–8.54	0.372			
EST	Yes	0.10	0.01–1.86	0.124	0.10	0.005–1.94	0.128
Prophylactic rectal NSAIDs use	Yes	5.21	0.61–44.7	0.133	4.55	0.49–42.6	0.184
RBO							
Type of NAT	GnP	0.74	0.17–3.25	0.685			
Preoperative waiting period	≥130 days	0.88	0.32–2.40	0.798			
FCSEMS diameter	6 mm	2.20	0.76–6.31	0.144	2.07	0.71–6.03	0.183
FCSEMS length	≥7 cm	2.03	0.73–5.69	0.177	1.91	0.67–5.43	0.226
Stent migration							
Reduction of the primary tumor	Yes	1.86	0.37–9.42	0.454			
FCSEMS placement before NAT	Yes	0.72	0.19–2.76	0.635			
FCSEMS diameter	6 mm	3.39	0.92–12.5	0.068			
FCSEMS length	≥7 cm	0.36	0.07–1.78	0.209			

Abbreviations: CI, confidence interval; EST, endoscopic sphincterotomy; FCSEMS, fully covered self‐expandable metal stent; GnP, gemcitabine plus nab‐paclitaxel; NAT, neoadjuvant therapy; NSAIDs, nonsteroidal anti‐inflammatory drugs; RBO, recurrent biliary obstruction.

## DISCUSSION

This study evaluated the efficacy and safety of PBD with 6‐mm diameter FCSEMSs in comparison with 10‐mm diameter FCSEMSs in patients with BR PC. Stent‐related NER (57% vs. 64%, *p* = 0.610), stent‐related AEs (4% vs. 15%, *p* = 0.263), overall RBO rates (39% vs. 23%, *p* = 0.168), cumulative incidence of RBO (HR, 2.24; 95% CI, 0.95–5.25; *p* = 0.065) and postoperative complications (9% vs. 10%, *p* > 0.999) were not significantly different between the two groups. Tumor involvement of the pancreatic duct was identified as a risk‐reducing factor for pancreatitis, while an FCSEMS diameter of 6 mm was not identified as a risk factor for RBO and stent migration.

In this study, we propose a new metric called the stent‐related NER, defined as the rate of completing surgery without developing preoperative events, which were in turn defined as RBO or stent‐related AEs. PBD is becoming more and more important, as NAT has become popular among patients with BR PC. As biliary AEs during NAT were reported to be associated with early postoperative recurrence and decreased overall survival,^18^ reducing not only RBO but also all other types of stent‐related AEs have become particularly important in the preoperative setting. We therefore believe that stent‐related NER is an important metric to consider for patients undergoing PBD.

With respect to stent‐related AEs, 6‐mm FCSEMSs may have an advantage over 10‐mm FCSEMSs. However, data are limited regarding the efficacy and safety of 6‐mm diameter FCSEMSs for PBD.^12^, ^13^ Kataoka et al.^12^ retrospectively compared 6‐mm FCSEMSs with plastic stents for PBD in 51 patients with PC. The incidence of RBO was significantly lower in the 6‐mm FCSEMS group (7.7% vs. 40.0%, *p* = 0.009), despite an insignificant but higher rate of pancreatitis (15.4% vs. 4.0%, *p* = 0.350). Surprisingly, stent migration was not observed in the 6‐mm FCSEMS group. The rate of RBO and stent migration in the 6‐mm group was higher in our study, probably due to the longer preoperative waiting period (111 days in our study vs. 38.5 days in the previous study^12^). Harai et al.^13^ retrospectively compared 6‐mm FCSEMSs with 10‐mm FCSEMSs in patients with PC. Subgroup analysis of patients with R/BR PC (*n* = 60) showed that the rate of pancreatitis tended to be lower in the 6‐mm group (3.8% vs. 25.0%, *p* = 0.08), while the non‐RBO rate was similar between the two groups (80.8% vs. 75.0%, *p* = 0.70). Differences in the non‐RBO rates between Harai's study^13^ and ours may also be attributable to differences in the preoperative waiting period. The preoperative waiting period was shorter in Harai's study^13^ (83 days each in the 6‐mm and 10‐mm group) compared to ours (111 days and 133 days in the 6‐mm and 10‐mm group, respectively), which may have resulted in the higher non‐RBO rates in both groups in their study.

As two of the seven patients who developed pancreatitis in the 10‐mm group experienced treatment delays in NAT and surgery, it was considered particularly important to avoid pancreatitis before surgery. On the other hand, slightly more stent migration was observed in the 6‐mm FCSEMS group, although the difference was not statistically significant (17% vs. 8%, *p* = 0.234). Differences in the rate of pancreatitis and stent migration may be attributable to differences in the mechanical properties of each SEMS.^19^, ^20^, ^21^ SEMSs with larger diameters may have higher axial force compared to their smaller counterparts, which is considered a risk factor for pancreatitis.^11^, ^22^ Smaller diameter SEMSs may have lower radial force, which is considered a risk factor for stent migration.^23^

One reason for the insignificant difference in stent‐related NER between the two groups (57% vs. 64%, *p* = 0.610) may be related to the rate of stent migration in the 6‐mm group. Development of SEMSs with new anti‐migration properties or the addition of anchoring plastic stents^24^, ^25^ may be useful to prevent stent migration and to improve the efficacy of 6‐mm FCSEMS.

This study has several limitations. First, this was a single‐center retrospective study with a limited sample size. Second, patients who underwent surgery had a longer preoperative waiting period than those diagnosed as unresectable because of the time between re‐staging after completion of NAT and the date of surgery. The preoperative waiting period was shorter in the 6‐mm group, which may be related to the proportion of patients who had FCSEMS placed after starting NAT and patients diagnosed as unresectable. Third, regimens of NAT were different between groups. The type of regimen may have differed in the rate of primary tumor size reduction, which may have affected the rate of stent migration. Finally, prophylactic rectal NSAIDs were more commonly administered in the 6‐mm group, which may have affected the rate of pancreatitis.

In conclusion, stent‐related NER, stent‐related AEs, overall RBO rates, and cumulative incidence of RBO were not significantly affected by FCSEMS diameter in patients undergoing NAT for BR PC. Further studies are needed to confirm the usefulness of 6‐mm diameter FCSEMS for PBD in patients with BR PC.

## CONFLICT OF INTEREST STATEMENT

Tsuyoshi Takeda and Takafumi Mie received honoraria from Boston Scientific Japan. Takashi Sasaki received honoraria from Boston Scientific Japan, Cook Medical Japan, and Century Medical.
